# PanSVR: Pan-Genome Augmented Short Read Realignment for Sensitive Detection of Structural Variations

**DOI:** 10.3389/fgene.2021.731515

**Published:** 2021-08-19

**Authors:** Gaoyang Li, Tao Jiang, Junyi Li, Yadong Wang

**Affiliations:** ^1^Center for Bioinformatics, School of Computer Science and Technology, Harbin Institute of Technology, Harbin, China; ^2^School of Computer Science and Technology, Harbin Institute of Technology, Shenzhen, China

**Keywords:** structure variation calling, pan-genome, read re-alignment, high-throughput sequencing data, repeat-rich region variation

## Abstract

The comprehensive discovery of structure variations (SVs) is fundamental to many genomics studies and high-throughput sequencing has become a common approach to this task. However, due the limited length, it is still non-trivial to state-of-the-art tools to accurately align short reads and produce high-quality SV callsets. Pan-genome provides a novel and promising framework to short read-based SV calling since it enables to comprehensively integrate known variants to reduce the incompleteness and bias of single reference to breakthrough the bottlenecks of short read alignments and provide new evidences to the detection of SVs. However, it is still an open problem to develop effective computational approaches to fully take the advantage of pan-genomes. Herein, we propose Pan-genome augmented Structure Variation calling tool with read Re-alignment (PanSVR), a novel pan-genome-based SV calling approach. PanSVR uses several tailored methods to implement precise re-alignment for SV-spanning reads against well-organized pan-genome reference with plenty of known SVs. PanSVR enables to greatly improve the quality of short read alignments and produce clear and homogenous SV signatures which facilitate SV calling. Benchmark results on real sequencing data suggest that PanSVR is able to largely improve the sensitivity of SV calling than that of state-of-the-art SV callers, especially for the SVs from repeat-rich regions and/or novel insertions which are difficult to existing tools.

## Introduction

Structural variants (SVs) are the genomic variations usually defined as genome rearrangement longer than 50 base pairs (bps), which alter a large number of bases in human genomes, although they are fewer than that of single nucleotide variants (SNVs) and short indels. Previous studies have demonstrated that there are many associations between SVs and human phenotypes and diseases (Weischenfeldt et al., 2013; Sudmant et al., 2015; Chiang et al., 2017), thus the comprehensive discovery of SVs in human genomes is fundamental to many genomics studies.

High throughput sequencing (HTS) technologies are rapidly developing and ubiquitously used in human genome re-sequencing projects. Especially, the short reads produced by mainstream platforms like Illumina sequencers play important roles to the detection of various types of genomic variations including SNVs, indels and SVs (Collins et al., 2019). Due to the high sequencing quality, short reads are feasible to call SNVs and indels and they have demonstrated their ability in many large-scale genomic studies to build the variation maps of various populations (Durbin et al., 2010; The 1000 Genomes Project Consortium, 2012; The UK 10K Consortium, 2015; Cong et al., 2021). However, due to the limited read length, short read had lower ability in SV calling theoretically and practically, comparing to that of the data produced by long reads sequencing platforms such as PacBio or ONT sequencers (Ebert et al., 2020; Beyter et al., 2021). For example, a previous study (Ebert et al., 2021) indicated that, on average 9,320 SVs per sample were called with short reads by three SV calling pipelines, however, this is still less than half of the number of SVs called by long reads. Many of SV calling tools designed for TGS long reads [for example sniffles (De Coster et al., 2019), cuteSV (Jiang et al., 2020), and svim (Heller and Vingron, 2019, 2020)], have the ability to call over 20,000 SVs per individual.” Therefore, it is important to develop novel approaches to improve the ability of SV calling with short reads since the sequencing cost of short reads is still much lower.

Many efforts have been made to develop short read-based SV calling approaches. Most of state-of-the-art SV callers [for example delly (Rausch et al., 2012), lumpy (Layer et al., 2014), manta (Chen et al., 2016), and CNVnator (Abyzov et al., 2011)] extract one or multiple kinds of signatures from read alignments, such as discordant read pair, split read, read depth, and local assembly, as evidences to detect SVs. However, all these kinds of signatures could be less effective in practice due to the shortcomings of read aligners which it is still non-trivial to produce the accurate and confident alignments around the breakpoints of SVs (Zook et al., 2020). Most of state-of-the-art read aligners, such as BWA-MEM (Li, 2013), NovoAlign, Bowtie2 (Langmead and Salzberg, 2012), and deBGA (Liu et al., 2016), use seed-and-extension approach. They usually neglect the highly repetitive seeds occurring many times in the reference, however, this could map the reads from repeat-rich regions incorrectly and further affect SV calling. Meanwhile, reads from long novel insertions cannot be correctly aligned in theory, since the abundance of the inserted sequences in reference. Thus, it could extract very few evidences for those insertion events from the alignment results.

With the increasing numbers of sequences samples and known genomic variations (Chaisson et al., 2019), pan-genome-based methods are promising to break through the bottlenecks to the alignment of short reads and provide new opportunities to solve the problems in SV calling. Pan-genome is the ensemble of all the genomes from a species (Sherman and Salzberg, 2020), and in practice it is usually composed by the genomes of multiple samples of the same species or a reference genome plus a set of genomic variations of a population. It has advantages to use a pan-genomes as reference instead of a single genome in read alignment since pan-genome enables to integrate much more reference information to help the alignment of SV-spanning reads. For example, with the integration of known SVs, pan-genome has less bias during the seeding process, so that aligners can locate reads to SV regions with more confidence. Moreover, the sequences of integrated SVs also help the aligners to implement full-length read alignments with high scores instead of the chimeric alignments with plenty of clippings, split alignments and discordant pairs under the circumstance of a single reference. Further, the alignments between reads and integrated SVs can also be used as the evidences of SVs in donor genomes.

However, it is still an open problem to well-organize pan-genome and take its advantage to implement effective and efficient read alignment and SV calling. Efforts have been made to the construction and organization of pan-genome (Sirén et al., 2011, 2020a; Paten et al., 2018; Rakocevic et al., 2019). Moreover, several read alignment and genotyping approaches have been proposed. VG (Garrison et al., 2018; Hickey et al., 2020), giraffe (Sirén et al., 2020b), minigraph (Li et al., 2020) are designed for aligning short reads and GraphAligner (Rautiainen and Marschall, 2020) is designed for aligning long reads. They show higher ability to read alignment and genotyping comparing to the traditional pipelines using single reference. However, most of them are not tailored to SV calling. Especially, these approaches still do not fully consider the divergences between known SVs and the SVs in donor genome, so that they could still have lowered ability to handle newly sequenced samples. Thus, novel computational approaches are still on demand. Moreover, the extraction and analysis of SV signatures is largely different between traditional and pan-genome-based approaches, and they could also be complementary to each other. However, it is also another open problem to integrate various approaches to achieve highest yields in SV calling tasks.

Herein, we propose a novel approach, i.e., Pan-genome augmented Structure Variation calling tool with read Re-alignment (PanSVR). PanSVR focuses to well-handle the potential SV-spanning reads under pan-genome framework to implement more sensitive SV calling. Mainly, it collects known SV information to build pan-genome SV reference and use it as anchors to precisely re-align chimeric reads and find the evidences of SVs with the improved alignments of the reads against pan-genome. Benchmark results on real sequencing data suggest that PanSVR enable to largely improve the sensitivity of SV calling than that of state-of-the-art SV callers, especially for the SVs from repeat-rich regions and/or novel insertions which are difficult to existing tools.

## Materials and Methods

### Overview of PanSVR Approach

The motivation of PanSVR is to take the advantages of known SVs as anchors to improve the sensitivity and accuracy of the alignment of SV-spanning reads to breakthrough the bottleneck of commonly used short read aligners. Moreover, with the improved read alignments, more homogeneous SV signatures can be captured and higher numbers of supporting reads can be found to facilitate the detection of SVs.

**FIGURE 1:**
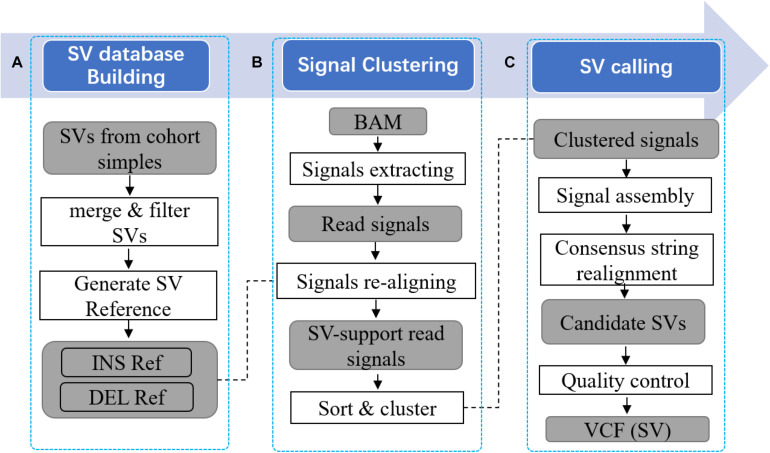
Overview of PanSVR SV calling process. Three main steps of PanSVR SV calling process. **(A)** In the first step, SV reference is built from known SVs; **(B)** In the second step, read signals are extracted from original BAM files and mapped to the SV reference; **(C)** Finally, read signals clustered around SV breakpoints are assembled and SV results generated from consensus strings.

Pan-genome augmented structure variation calling tool with read re-alignment uses several tailored methods to implement this approach. Mainly, it is composed by two parts. Firstly, PanSVR integrates known SVs into commonly used reference genome to build an augmented pan-genome SV reference. The SV reference consists of the sequences around SV sites including the sequences of novel insertions which do not exist in the original reference. This reference is used as anchors to provide additional information for read aligners to improve the reads having clippings, split alignments or discordantly placed which are potentially SV-spanning reads. Secondly, PanSVR collects potential SV-spanning reads and employs short read aligner to re-align those reads against the SV reference. The newly supplied alignments have fewer large divergences such as clippings and split-alignments but more homogenous and confident alignments with the anchors, i.e., the sequences around SV sites. Thus, more homogeneous SV evidences can be collected by PanSVR to further use them to infer accurate SV events. Mainly, PanSVR approach have three main steps as following (Figure 1).

(1) Given a set of known SV events (in VCF format), PanSVR converts each of them as an anchor sequence. The generated anchor sequences are then concatenated to build the SV reference and further being indexed by a de Bruijn graph-based genome indexing (RdBG-index) approach (Liu et al., 2016).
(2) Given a set of aligned sequencing reads (in BAM/CRAM format), PanSVR extract the reads having SV signatures (such as clippings and split alignments) and re-align them against the SV reference with the help of RdBG-index and a tailored realignment method. The results are filtered based on the new and original alignments of the same reads and PanSVR clusters them based on their mapping coordinates.
(3) PanSVR separately assemble the reads for all the clusters to generate consensus sequences. Each of the generated sequence is precisely aligned to local region around SV sites in the original reference. The alignment results are used as evidences to infer SVs.

### The Construction of SV Reference

Initially, an SV related pan-genome reference (“SV reference”) is built from known SVs. Using a reference and a set of SVs records in VCF format as inputs, PanSVR extracts the sequences around the breakpoints of known SVs and stores them in a FASTA format file. It is also worth noting that the current version of PanSVR accepts only one VCF file to build SV reference. However, SV merging tools like SURVIVOR (Jeffares et al., 2017) are feasible to merge multiple SV sets before the construction of SV reference. By default, the sequences of 250 bp flanking SV breakpoints are extracted to construct SV reference as they are long enough to align the short reads produced by mainstream platforms. In details, PanSVR constructs SV reference by the following methods:

(1) For each of the deletions, genomic sequences upstream the first breakpoint and downstream the second breakpoint are directly concatenated together to make the SV anchor sequence;
(2) For each of the insertions and duplications, the inserted (or duplicated) sequences recorded in the ALT field of VCF file are extracted, and the SV anchor sequence is produced by concatenating the local reference sequence upstream the breakpoint, the inserted sequences and the local reference sequence downstream the breakpoint.

Structure variation reference is generated by concatenating all the generated SV anchor sequences. Further, PanSVR employs a de Bruijn graph-based indexing approach to index SV reference (the default value of k-mer is 22 bp) for the realignment of potential SV-spanning reads.

### The Realignment and Clustering of Potential SV-Spanning Reads

Pan-genome augmented structure variation calling tool with read re-alignment recognizes the reads potentially spanning SV sites according to their alignments against original reference, and realigns them against the SV reference. Especially, the reads are handled by two steps, i.e., single end read mapping and mate pairing. Further, the realigned reads are clustered by their coordinates and SV signals for SV inference. The method is implemented in four sub-steps as following.

#### Chimeric Reads Extraction

Reads with chimeric alignments are initially extracted from original SAM/BAM/CRAM files and stored as FASTQ format. Pair-end reads are re-paired by their names if the input file is sorted BAM/CRAM file. In details, PanSVR rejects the read-pairs being perfectly aligned to the reference, i.e., no more than one mismatch for any end in a read-pair and other reads are extracted. This is a restrict condition since SNPs and indel are also useful for SV detection if the reads are mapped to highly repetitive regions, such as VNTRs or STRs. The alignment information related to SV calling is extracted, including alignment position, alignment score, CIGAR, MAPQ, and ISIZE if available. The information is further recorded in the comment field of the converted FASTQ file.

#### Single-End Read Realignment

**FIGURE 2:**
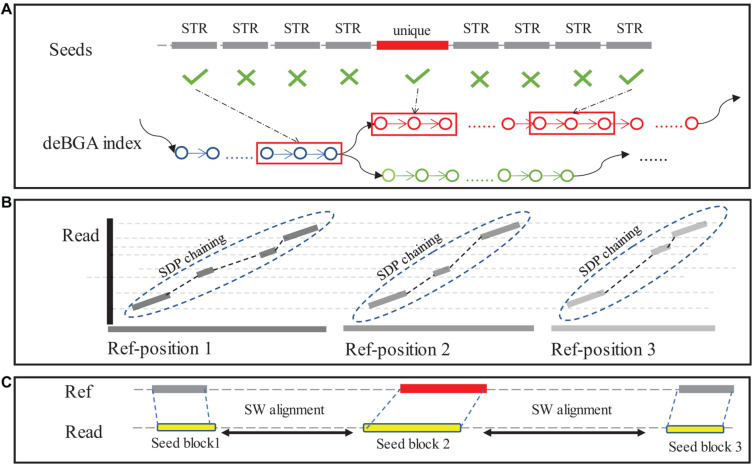
The seeding-chaining-and-extension in the alignment step. **(A)** The seeds generated in “unique region” of reads are located in reference using deBGA index. **(B)** Seeds within UNITIG of deBGA index will be greedy chained to longer blocks, then those blocks will be mapped to reference and chained again using SDP. **(C)** Sequence between chained blocks will be aligned using NW algorithm.

The extracted reads are re-aligned to SV reference using a seeding-chaining-and-extension approach (Figure 2). To reduce computational cost, PanSVR selects unique k-mers in a read as seeds (default value of k: 20), unlike traditional seeding methods. This design is to handle repetitive k-mers within STR or VNTR regions which could appear hundreds and thousands of times in reference and consume plenty of time during the seeding and chaining process. Other than unique seeds, the seeds from repetitive regions are also employed, if they are placed at either end of the reads (Figure 2A).

A two-phase chaining method is used for chaining the seeds. In the first phase, seeds are chained within the unitigs of RdBG-index of SV reference to generate longer match blocks from the shorter seeds. The match blocks are then mapped back to original reference as long seeds. If a match block is highly repetitive, i.e., it can be mapped to over 1000 genomic positions, 1000 positions are randomly selected for further processing. In the secondphase, the long seeds are chained by using a sparse dynamic programming (SDP)-based method with following functions:

(1) $$f\left(LS_{p}\right)=\text{max}\left\{\max_{p>q\geq 1}\left\{f\left(LS_{q}\right)+\text{L}\left(LS_{pq}\right)-\theta \left(p,q\right)\right\},L\left(LS_{p}\right)\right\}$$

(2) $$\theta \left(p,q\right)=0.125\times \left(\left(LS_{p}^{R}-LS_{q}^{R}\right)-\left(LS_{p}^{r}-LS_{q}^{r}\right)\right)+3$$

where LS_*p*_ and LS_*q*_ are the p-th and q-th long seeds (sorted by coordinates in reference); L(*LS*_*p*_) is the length of long seed p and L(*LS*_*pq*_) is the length of LS_*p*_ (only consider the part that not overlap with LS_*q*_). $LS_{p}^{r}$ is the position of LS_*p*_ on the reference, and $LS_{p}^{r}$ is the position of LS_*p*_ on the read; *f*(LS_*p*_) is the scoring function for the LS_*p*_, and θ(*p*, *q*) is the penalty score for the two chained long seeds LS_*p*_ and LS_*q*_.

In extension step, a traditional Smith-Waterman alignment is implemented for the top 12 seed chains with highest scores using ksw2 library (Li, 2018; Suzuki and Kasahara, 2018). The results are recorded in a list as single end alignment.

#### Mate Read Pairing

For a read pair, PanSVR uses the single end alignments for the both two ends of a read pair and their original alignments to compose a concordant pair-end alignment and compute the score of the refined alignment. Since the coordinates in the SV reference are not always same to the coordinates in original reference, the two coordinates of the original alignments could be divided to two different values that one of them is not changed and the other is adjusted by the length of the corresponding SV. Both two the values can be used as its coordinates. The score of a read pair is defined as the sum of alignment scores for both ends. When the two ends in a pairing condition have right directions and the ISIZE is within 1.5 times standard deviations of mean ISIZE, an additional score is added. The final score of a read pair is calculated using the following functions:

(3) $$S\left(RP_{i}\right)=\max_{\begin{array}{c} N_{i}>p\geq 1\\ M_{i}>q\geq 1 \end{array}}\left\{s\left(R1_{p}\right)+\text{s}\left(\text{R}2_{q}\right)+\theta \left(p,q\right)\right\}$$

(4) $$\theta \left(p,q\right)=\left\{ \begin{array}{cc} \text{K} & ifR1_{p}paiedwith{\mathrm{R2}}_{q}properly\\ 0 & otherwise \end{array}\right.$$

where *S*(*RP*_*i*_) is the final score of the i-th read pair; *N*_*i*_ is the number of single end alignment results for the first read in read pair and *M*_*i*_ is the number for results for the second read; *s*(*R*1_*p*_) is the score of the p-th single end alignment result for first read in a read pair, and *s*(R2_*q*_) is the score of the q-th single end alignment result for the second read in that read pair; θ(*p*, *q*) is the additional score be added when the two results pairing properly.

All pairing conditions are sorted by the scores and the one with the highest score is output as paired alignment result. It is also worth noting that the alignment result is discarded and the corresponding read-pair is recorded as unmapped if its alignment result (or one of the multiple results with equal scores) is not made by PanSVR but the original aligner. All the remaining alignment results are stored in SAM format. An additional tag that records the ID of SV anchor sequence is added in the SAM optional field, and it will be used to cluster the read in the following steps.

#### Read Clustering

All the SAM records of the improved alignments are sort by their positions in the SV reference. Since there could be multiple known SVs in highly repetitive regions and some of various known SVs could overlap with each other, the chimeric reads could be mistakenly assigned during read clustering. To address this issue, PanSVR clusters nearby known SVs as a group and only keeps the top two SVs with highest number of supporting reads and the reads assigned to other nearby SVs are re-assigned to them. Herein, the SVs are clustered in a greedy manner, i.e., an SV is added to a cluster if its upstream breakpoint is within 50 bp of the downstream border of the cluster, and the cluster expends until no nearby SV can be added into it. For a cluster, PanSVR separately counts the numbers of the reads being aligned to the SVs and uses these numbers as the scores of the SVs. For the reads not in the top two clusters, each of them is re-assigned to one of the two SVs by a simple k-mer counting method. That is, PanSVR counts the numbers of identical k-mers between a read and the anchor sequences of the two SVs and re-assign the read to the SV with more identical k-mers. If the two SVs have equally high numbers, the read is randomly assigned.

### The Assembly of Clustered Read and the Inference of SVs

Pan-genome augmented structure variation calling tool with read re-alignment implement an assembly for each of the clusters to produce the consensus sequence of the reads. The generated sequences are then aligned to the SV reference and PanSVR collects SV evidences from the alignment results. The method is implemented in four sub-steps as following.

#### Read Preprocessing

Pan-genome augmented structure variation calling tool with read re-alignment does a filtration on the reads before assembly with two rules to reduce false positives. Firstly, a proportion of reads with low scores are filtered out from the SV reference regions having extremely high read coverages. More precisely, PanSVR partitions a given reference region into 64 bp blocks and calculates the read coverages of the blocks. If a block has 1.5 times or higher read depth than average read depth, the reads having low scores in the block are discarded. Secondly, the reads are filtered by mapping quality. That is, for a given cluster, if over 80% of the reads have MAPQ = 0 for their original alignments and the scores of their improved alignments produced by PanSVR are also close to that, the read cluster is considered as an uncertain cluster and being discarded.

#### Assembly of Clustered Reads

Pan-genome augmented structure variation calling tool with read re-alignment uses a modified version of the assembly module of MANTA (Chen et al., 2016) to implement read assembly for all the clusters. Moreover, if a cluster belongs to a long SV region, i.e., the length of the corresponding SV is over 500 bp, the SV region is partitioned into 500 bp blocks and the assembly is separately implemented for the blocks. When the employed assembler picks up a read to extend the contig, it records at which position the read joins in the assembling contig. This information guides the realignment of the reads to the contig after assembly. Only mismatches are allowed in the realignment of reads to contig. Read coverage information on consensus sequence is calculated based on the realignment results.

#### Alignment of Consensus Sequence

For a consensus sequence, PanSVR detects some candidate positions in SV reference to implement local alignment at first. These candidate positions are from the mapping positions of the supporting reads in SV reference with some additional filtrations. Firstly, if all the candidate positions are out of range, the consensus sequence is discarded. Secondly, at least one read used in the generation of the consensus sequence should have a realignment score higher than that or its original alignment. After the filtration, a Needleman-Wunsch alignment is implemented for each of the candidate positions. The mismatches and indels between the consensus sequence and local sequence in SV reference is recorded at each position of SV reference. Moreover, read depths along the consensus sequences are also stored by all the corresponding coordinates in SV reference.

#### SV Calling and Genotyping

Pan-genome augmented structure variation calling tool with read re-alignment infers SVs from the alignment of consensus sequences. A candidate SV other than novel insertions implied by a consensus sequence is inferred if it has high enough depth at both the two breakpoints. Meanwhile, for novel insertions, the inserted sequence should also have high depth. Moreover, the positions of breakpoints and the inserted sequences are adjusted by the variations to make a more accurate inference. After the adjustment, SVs longer than 50 bp are kept and further genotyped with the coverage information.

## Results

### Implementation of Benchmark

To assess the ability of PanSVR, we composed an SV reference with a set of high-quality SVs at first. Mainly, the SVs are derived from PacBio CCS datasets of 16 different samples. Thirteen of them are from Human Genome Structural Variation Consortium (HGSVC) database where the datasets are phased assembly of CCS reads. There are two haplotypes of for each of the samples, and we aligned those genomes against human reference genome (version: hs37d5) by minimap2 and input the alignments into SVIM-asm (Heller and Vingron, 2019, 2020) to produce SV callsets. Moreover, we also downloaded three SV callsets of Genome in a Bottle (GIAB) Trio samples HG002, HG003, and HG004. These callsets are produced by GIAB consortium from PacBio CCS datasets using PBSV pipeline. SVs from different samples were merged by the following rule: two SVs were merged if they were of the same type and their breakpoints were within 50 bps. The merge operation was implemented by using SURVIVOR (Jeffares et al., 2017).

We benchmarked PanSVR on three real sequencing datasets produced by Illumina platforms from various samples (i.e., HG00512 and HG002) with various read lengths (i.e., 126, 148, and 250 bp). Refer to Supplementary Table 2 for more detailed information. Two state-of-the-art short read-based SV callers, i.e., Manta and Delly, were also implemented on the same datasets for comparison. During the benchmark, leave-one-out strategy was applied for PanSVR, i.e., the SVs of the corresponding sample was removed from the known SV sets beforehand so that the constructed SV reference is blind to the benchmarked dataset. The reads were aligned against human reference hs37d5 by BWA-MEM with default settings. Manta and Delly directly detected SVs from the read alignments.

### Results on Real Sequencing Datasets

The sensitivity, accuracy and F1-score of the benchmarked SV callers were assessed by using the “merge” and “genComp” commands of SURVIVOR. All the benchmarks were carried out on an Ubuntu Linux server with one AMD 3950X CPU (32 cores) and 256 GB RAM. All the SV callers were run in using 8 CPU threads. Mainly, three issues were observed from the results.

#### PanSVR Has Good SV Calling Yields

**FIGURE 3:**
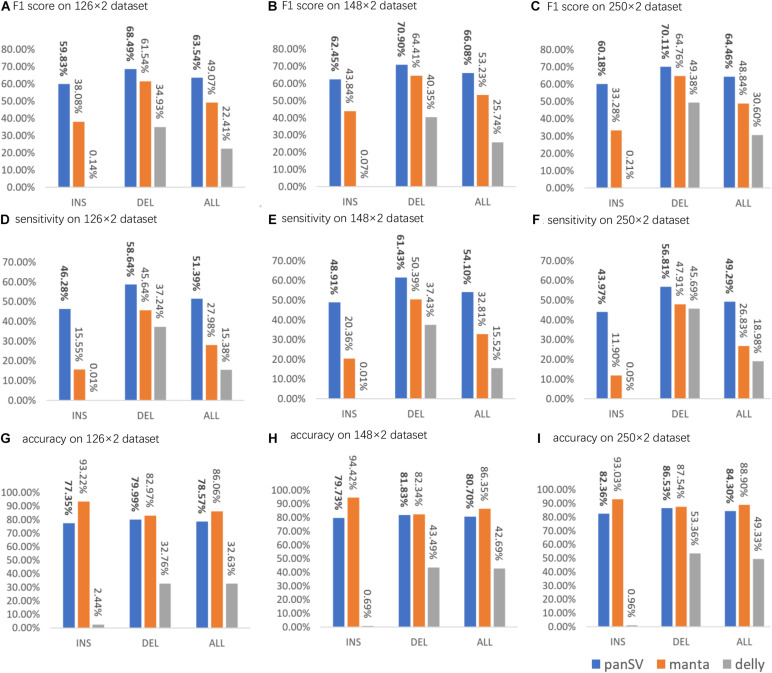
The sensitivity, precision and F1 score of PanSVR, manta and delly on three NGS datasets. **(A,D,G)** F1 score, sensitivity and accuracy of SV calling results of PanSVR, manta and delly applied on Illumina 126 × 2 dataset; **(B,E,H)** The SV calling results of those tools on Illumina 148 × 2 dataset; **(C,F,I)** The SV calling results of those tools on Illumina 250 × 2 dataset.

For all the datasets, PanSVR obviously outperformed Manta and Delly for F1-scores on both insertions and deletions (Figure 3). We investigated the intermediate results of PanSVR and found that the SV reference greatly helped to improve the alignment of SV-spanning reads. Although the known SV sets cannot cover all the SVs of the testing samples, the anchor sequences of the SV reference enable to rescue many reads which cannot be correctly and/or confidently aligned with the original reference. This feature largely improves the sensitivity of SV calling, especially for large insertions. For all the datasets, the numbers of insertions detected by PanSVR are nearly two times to that of Manta. Moreover, Delly showed a relatively poor ability to detect insertions, i.e., it only called a few hundreds of insertions for each sample and only a few of them were true positive. It is also worth noting that all the callers have relatively good results on deletions since short reads spanning deletions are much easier to be aligned and the SV signatures of short reads around deletion events, such as discordant read pairs and split alignments, are less complicated and more homogeneous.

As for the influence of read length on the SV calling ability, most of time, longer reads do help to achieve better SV calling results. For Delly, the F1 scores increased with the increase of read length and reach best value on the 250 bp dataset, while PanSVR and manta achieved best F1 scores on 148 bp dataset. We investigated the details of the results and found that the large numbers of low-quality bases at the tails of the 250 bp reads affected the local assembly operation of PanSVR.

#### PanSVR Has Good Ability to Call Long Insertions

**FIGURE 4:**
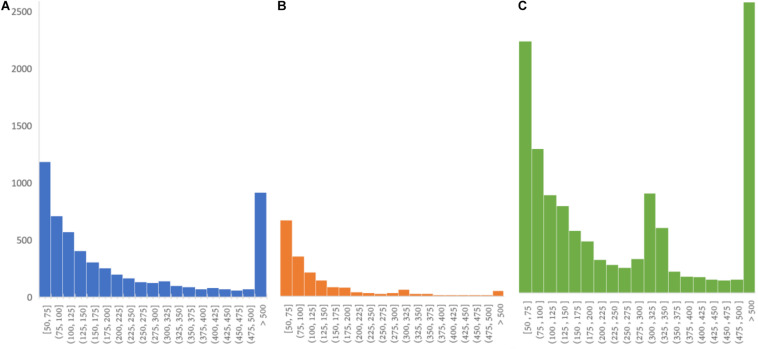
SV length distribution of insertions from HG00512 126 × 2 sample. **(A)** Insertion length distribution of PanSVR true positive set (917 insertions longer than 500 bp); **(B)** Insertion length distribution of manta true positive set (48 insertions longer than 500 bp); **(C)** Insertion length distribution of CCS SV set (2,660 insertions longer than 500 bp).

It is a still non-trivial task for state-of-the-art short read-based callers to detect long insertions due to two issues. First, when an insertion is longer than the read length, one or two ends of a read pair around the insertion could be unmapped. Second, the length of insertion cannot be easily estimated and assembling all reads around and within an insertion is usually hard. Based on pre-built SV reference, PanSVR enable to detect long insertions with the help of SV anchor sequences that the reads can be effectively realigned to imply plenty of SV signatures. Moreover, PanSVR also has the ability to detect the SNVs and indels within the inserted strings of the sequenced sample from the realignments of the reads, so that the inserted sequences of donor samples can be correctly recovered even if they are divergent to the anchor sequences of SV reference. As showed in Figure 4, there are only 48 > 500 bp true positive insertions in the callset of Manta, and the corresponding number for PanSVR is 917. However, we also observed that PanSVR has lowered ability to handle ALU insertions (as show in Figure 4A which the length distribution of the SVs detected by PanSVR has no significant peak around 300 bp). This is mainly due to that ALUs are extremely repetitive in human reference genome and the average mapping quality of the reads being aligned to ALU regions are usually close to 0. PanSVR filters out such regions according to the low MAPQ so that ALU insertions could be missed.

#### The Ability of PanSVR Could Be Complementary to State-of-the-Art SV Callers

Most of existing SV callers use chimeric alignments such as split reads, discordant read pair and large clippings as SV signatures. PanSVR does not rely on those kinds of signatures but use a different approach, so that it could produce higher-quality SV callsets by merging the results of PanSVR and other tools. We merged the results of PanSVR and Manta using SURVIVOR by various parameters. Firstly, we generated the union SV calling set of PanSVR and Manta. The SVs are treated as one when their breakpoints are distanced less than 50 bp. For all the samples and SV types, the merged SV callset achieved higher sensitivities and F1-scores than the callsets separately produced by PanSVR and Manta (Supplementary Table 3), although the precisions could decrease. For example, the merged callset of the 148 bp HG002 dataset called 12272 true positive SVs with 77.4% true positive rate, while PanSVR and Manta called 11540 and 6980 true positive SVs, respectively. We also tried to generate an intersection SV set from the results of the two tools. It reached more than 96% true positive rate in all three datasets, however, the F1-score slightly decreased comparing to that of Manta only (Supplementary Table 4).

We also assessed the speed and memory footprint of PanSVR. It takes less than 2.7 h to process all steps using 8 threads for a 60x coverage dataset. This is slower than Manta and Delly, but still affordable. This is mainly due to the realignment procedure of the approach which is more computation-intensive than that of directly analyzing the read alignment results like most of short read-based SV callers do. Moreover, the time cost of the assembly of clustered reads is also non-neglectable. However, PanSVR still has good scalability since all the steps can be run in a parallel way. The memory footprint of the PanSVR is about 3.5 GB in the benchmark where the memory is mainly used by the RdBG-index of SV reference in the read realignment step.

## Discussion

Previous studies (Hickey et al., 2020; Sirén et al., 2020b) have demonstrated that ability of pan-genomes to help the alignment of short reads and SNP/INDEL calling. In this study, we introduce a pan-genome augmented read realignment and SV calling tool, PanSVR. Results on real NGS datasets demonstrate that it is feasible to use pan-genome based realignment approach to realign short reads to break through the bottleneck of short read alignment and further improve SV calling.

Mainly, we found that two main categories of SVs can be better handled with the pan-genome-based method. Firstly, the SVs in tandem repeat regions can be recused by PanSVR. This is due to that SNPs and INDELs within VNTR or STR can be used to correct short read alignments around those regions. A case is shown in Supplementary Figure 1 that a 70 bp insertion around chr1:1913259 were successfully called by PanSVR, however, no other tool is able to detect them in the benchmark. We checked alignment results around those regions manually and found that the spanning reads can be fully mapped to that region by BWA-MEM, but with a number of mismatches and indels. The lower quality alignments affect the callers and the SVs are recognized as multiple SNP/indels. However, these reads can be re-aligned with exact matches to the SV reference by PanSVR and evidences can be collected to call the SVs confidently.

Secondly, the results indicated that pan-genome-based method greatly help to improve recall of long insertions which is surprising since additional reference information is added. It is shown that PanSVR has a nearly 20 times higher number of long insertion (>500 bp) calls than that of Manta. This is very complementary to the state-of-the-art SV calling approaches. A case is shown in Supplementary Figure 2 that a 955 bp insertion at chr2:235423389, which cannot be called by other callers but PanSVR. The read alignments show that there are few split-read and discordant read pair signals around the SV breakpoints, so that the SVs are hard to detect, however, the realignment against the SV reference recused most of SV-spanning reads and provided homogeneous SV signatures.

The results also suggest that it is also helpful to merge the SV callsets by multiple callers to further increase sensitivity. For example, the sensitivity increased by 3.4% for the 148 bp dataset comparing to that of the original callset of PanSVR. This is consistent with previous studies (Chaisson et al., 2019) as multiple tools could be complementary to each other by various kinds of signatures and models. However, it is also worth noting that the simple union of the callsets of various tools could introduce more false positives, so that more advanced approaches for the filtration and prioritization of SV calls are still needed.

There is still a huge gap for the sensitivity of SV calling between short and long sequencing reads, although pan-genome is used. There could be caused by two issues.

Firstly, some of the SVs in donor genomes are individually specific and their breakpoints are far away from known SVs or even not related to them at all. In this situation, the pan-genome-based method cannot provide much help and the detection of SV only depends on the alignments of the reads against original reference. A case is shown in Supplementary Figure 3 that an 87 bp insertion in chr1:2213294 is unique for HG002 sample. It was not in the SV reference during the leave-one-out benchmark and PanSVR failed. However, with the many on-going population-scale genomics studies, it is promising to build more comprehensive SV databases. For example, a recent study (Beyter et al., 2021) has built an SV database of Iceland population with 133,886 reliably genotyped SVs and such SV databases could be available for various populations with the ubiquitous application of high-throughput sequencing technologies.

Secondly, the limited length of short reads is still a bottleneck even if under the circumstance of pan-genome. Especially, this could cause lower coverage to correct anchors in SV reference for PanSVR. A case is shown in Supplementary Figure 4 that there is nearly no read being aligned to a 103 bp insertion in chr1:1855662. The inserted sequence is highly repetitive, i.e., ACCACCCCCCAGCTCACAGCCCACCCCCCCATCTCACCG CCCAGCCCCCCCATCTCACCAGCTGCCCCCTCCCGGGCA CACCGCCCACCCCCCCATCTCACCA. Such repeats can still not be spanned by short reads and the reads are usually mapped to other copies of the sequences with nearly perfect alignments, i.e., exactly matched without mismatch or indel. In this situation, the SV is non-trivial to be solved. Moreover, the results also indicated that PanSVR could make false positives in some cases. We checked the SVs mistakenly called by PanSVR and found that they were mainly in repeat regions. Some consensus sequences were not long enough to across the repeat region, either. Wrong alignment of them might cause wrong SV calling.

Pan-genome-based SV calling approach is promising to the comprehensive discovery of individual genomes, especially for short read datasets. With the supplement of additional SV information, it enables to produce higher-quality alignments and help to provide more evidences to make SV calls with confidence. However, there are still open problems to the use of known SVs, moreover, some of SVs can still not solved with the available SV databases. These are also important future works to us to further improve PanSVR approach. With the higher sensitivity and yield, we believe that PanSVR has the potential to many genomics studies.

## Data Availability Statement

The original contributions presented in the study are included in the article/Supplementary Material, further inquiries can be directed to the corresponding authors.

## Author Contributions

GL and TJ designed the method. GL implemented the method. GL and JL performed the analysis. All authors wrote the manuscript.

## Conflict of Interest

The authors declare that the research was conducted in the absence of any commercial or financial relationships that could be construed as a potential conflict of interest.

## Publisher’s Note

All claims expressed in this article are solely those of the authors and do not necessarily represent those of their affiliated organizations, or those of the publisher, the editors and the reviewers. Any product that may be evaluated in this article, or claim that may be made by its manufacturer, is not guaranteed or endorsed by the publisher.
