# Supplementary material for: PanSVR: Pan-Genome Augmented Short Read Realignment for Sensitive Detection of Structural Variations
Source: Front Genet. 2021 Aug 19;12:731515. doi: 10.3389/fgene.2021.731515 (PMC8417358; doi:10.3389/fgene.2021.731515)
Supplement: Supplementary file 1 [file Data_Sheet_1.docx]

Supplementary Material

[**Supplementary Table 1. The samples used to build SV reference 2**](#_Toc75714001)

[**Supplementary Table 2. The real NGS reads used in benchmark. 4**](#_Toc75714002)

[**Supplementary Table 3. Union call set of panSV and manta on HG002 148 dataset 4**](#_Toc75714003)

[**Supplementary Table 4. Intersection call set of panSV and manta on HG002 148 dataset 4**](#_Toc75714004)

[**Supplementary figure 1. The IGV result of a 70 bp insertion around chr1:1913259 5**](#_Toc75714005)

[**Supplementary figure 2. The IGV result of a 955 bp insertion around chr2:235423389 6**](#_Toc75714006)

[**Supplementary figure 3. The IGV result of an 87 bp SV around chr1: 2213294 7**](#_Toc75714007)

[**Supplementary figure 4. The IGV result of a 103 bp SV around chr1: 1855662 8**](#_Toc75714008)

[**Command lines used in benchmark implementation. 9**](#_Toc75714009)

1. **The samples used to build SV reference**

| **#** | **isolate** | **cohort** | **gender** | **Sample relation-ship** | **download link** | |
| --- | --- | --- | --- | --- | --- | --- |
| 1 | HG00512 | Han Chinese South, East Asian Ancestry | Male | No relation | | http://ftp.1000genomes.ebi.ac.uk/vol1/ftp/data_collections/HGSVC2/release/v1.0/assemblies/20200628_HHU_assembly-results_CCS_v12/assemblies/phased/v12_HG00512_hgsvc_pbsq2-ccs_1000-pereg.h1-un.racon-p2.fasta |
| 2 | HG00513 | Han Chinese South, East Asian Ancestry | Female | No relation | | http://ftp.1000genomes.ebi.ac.uk/vol1/ftp/data_collections/HGSVC2/release/v1.0/assemblies/20200628_HHU_assembly-results_CCS_v12/assemblies/phased/v12_HG00513_hgsvc_pbsq2-ccs_1000-pereg.h1-un.racon-p2.fasta |
| 3 | HG00731 | Puerto Rican in Puerto Rico, American Ancestry | Male | father of HG00733 | | http://ftp.1000genomes.ebi.ac.uk/vol1/ftp/data_collections/HGSVC2/release/v1.0/assemblies/20200628_HHU_assembly-results_CCS_v12/assemblies/phased/v12_HG00731_hgsvc_pbsq2-ccs_1000-pereg.h1-un.racon-p2.fasta |
| 4 | HG00732 | Puerto Rican in Puerto Rico, American Ancestry | Female | mother of HG00733 | | http://ftp.1000genomes.ebi.ac.uk/vol1/ftp/data_collections/HGSVC2/release/v1.0/assemblies/20200628_HHU_assembly-results_CCS_v12/assemblies/phased/v12_HG00732_hgsvc_pbsq2-ccs_1000-pereg.h1-un.racon-p2.fasta |
| 5 | HG00733 | Puerto Rican in Puerto Rico, American Ancestry | Female | daughter of HG00731 and HG00732 | | http://ftp.1000genomes.ebi.ac.uk/vol1/ftp/data_collections/HGSVC2/release/v1.0/assemblies/20200628_HHU_assembly-results_CCS_v12/assemblies/phased/v12_HG00733_hgsvc_pbsq2-ccs_1000-pereg.h1-un.racon-p2.fasta |
| 6 | HG02818 | Gambian in Western Division, The Gambia - Mandinka, African Ancestry | Female | No relation | | http://ftp.1000genomes.ebi.ac.uk/vol1/ftp/data_collections/HGSVC2/release/v1.0/assemblies/20200628_HHU_assembly-results_CCS_v12/assemblies/phased/v12_NA12878_giab_pbsq2-ccs_1000-pereg.h1-un.racon-p2.fasta |
| 7 | HG03125 | Esan in Nigeria, African Ancestry | Female | No relation | | http://ftp.1000genomes.ebi.ac.uk/vol1/ftp/data_collections/HGSVC2/release/v1.0/assemblies/20200628_HHU_assembly-results_CCS_v12/assemblies/phased/v12_NA19238_hgsvc_pbsq2-ccs_1000-pereg.h1-un.racon-p2.fasta |
| 8 | HG03486 | Mende in Sierra Leone, African Ancestry | Female | No relation | | http://ftp.1000genomes.ebi.ac.uk/vol1/ftp/data_collections/HGSVC2/release/v1.0/assemblies/20200628_HHU_assembly-results_CCS_v12/assemblies/phased/v12_NA19239_hgsvc_pbsq2-ccs_1000-pereg.h1-un.racon-p2.fasta |
| 9 | NA12878 | Western European ancestry | Female | No relation | | http://ftp.1000genomes.ebi.ac.uk/vol1/ftp/data_collections/HGSVC2/release/v1.0/assemblies/20200628_HHU_assembly-results_CCS_v12/assemblies/phased/v12_NA19240_hgsvc_pbsq2-ccs_1000-pereg.h1-un.racon-p2.fasta |
| 10 | NA19238 | Yoruba in Ibadan, Nigeria, African Ancestry | Female | mother of NA19240 | | http://ftp.1000genomes.ebi.ac.uk/vol1/ftp/data_collections/HGSVC2/release/v1.0/assemblies/20200628_HHU_assembly-results_CCS_v12/assemblies/phased/v12_NA24385_hpg_pbsq2-ccs_1000-pereg.h1-un.racon-p2.fasta |
| 11 | NA19239 | Yoruba in Ibadan, Nigeria, African Ancestry | Male | father of NA19240 | | http://ftp.1000genomes.ebi.ac.uk/vol1/ftp/data_collections/HGSVC2/release/v1.0/assemblies/20200717_HHU_assembly-results_CCS_v12/assemblies/phased/v12_HG02818_hgsvc_pbsq2-ccs_1000-pereg.h1-un.racon-p2.fasta |
| 12 | NA19240 | Yoruba in Ibadan, Nigeria, African Ancestry | Female | daughter of NA19238 and NA19239 | | http://ftp.1000genomes.ebi.ac.uk/vol1/ftp/data_collections/HGSVC2/release/v1.0/assemblies/20200717_HHU_assembly-results_CCS_v12/assemblies/phased/v12_HG03125_hgsvc_pbsq2-ccs_1000-pereg.h1-un.racon-p2.fasta |
| 13 | NA24385 | Ashkenazi Jewish | Male | No relation | | http://ftp.1000genomes.ebi.ac.uk/vol1/ftp/data_collections/HGSVC2/release/v1.0/assemblies/20200717_HHU_assembly-results_CCS_v12/assemblies/phased/v12_HG03486_hgsvc_pbsq2-ccs_1000-pereg.h1-un.racon-p2.fasta |
| 14 | NA24631 | Han Chinese South, East Asian Ancestry | Male | son of NA24694 and NA24695 | | https://ftp-trace.ncbi.nlm.nih.gov/giab/ftp/data/ChineseTrio/analysis/PacBio_pbsv_05212019/HG005_hs37d5.pbsv.vcf.gz |
| 15 | NA24694 | Han Chinese South, East Asian Ancestry | Male | father of NA24631 | | https://ftp-trace.ncbi.nlm.nih.gov/giab/ftp/data/ChineseTrio/analysis/PacBio_deepvariant_gatk_pbsv_08282020/HG006/HG006.hs37d5.pbsv.vcf.gz |
| 16 | NA24695 | Han Chinese South, East Asian Ancestry | Female | mother of NA24631 | | https://ftp-trace.ncbi.nlm.nih.gov/giab/ftp/data/ChineseTrio/analysis/PacBio_deepvariant_gatk_pbsv_08282020/HG007/HG007.hs37d5.pbsv.vcf.gz |

1. **The real NGS reads used in benchmark.**

| **#** | **isolate** | **read length** | **sequencing method** | **download link** |
| --- | --- | --- | --- | --- |
| 1 | HG00512 | 126X2 | Illumina HiSeq 2500 | http://ftp.1000genomes.ebi.ac.uk/vol1/ftp/data_collections/hgsv_sv_discovery/data/CHS/HG00512/high_cov_alignment/ |
| 2 | HG002 | 148X2 | Illumina HiSeq 2500 in Rapid mode (v1) | https://ftp-trace.ncbi.nlm.nih.gov/giab/ftp/data/AshkenazimTrio/HG002_NA24385_son/NIST_HiSeq_HG002_Homogeneity-10953946/ |
| 3 | HG002 | 250X2 | Illumina HiSeq 2500 in Rapid mode(v2) | https://ftp-trace.ncbi.nlm.nih.gov/giab/ftp/data/AshkenazimTrio/HG002_NA24385_son/NIST_Illumina_2x250bps/reads/ |

1. **Union call set of panSV and manta on HG002 148 dataset**

|  | **126×2 Illumina** | | | **148×2 Illumina** | | | **250×2 Illumina** | | |
| --- | --- | --- | --- | --- | --- | --- | --- | --- | --- |
|  | **Sensitivity** | **Precision** | **F1 score** | **Sensitivity** | **Precision** | **F1 score** | **Sensitivity** | **Precision** | **F1 score** |
| **INS** | 47.61% | 77.09% | 60.58% | 50.76% | 79.48% | 63.52% | 45.67% | 82.13% | 61.24% |
| **DEL** | 63.67% | 74.48% | 68.86% | 67.03% | 75.23% | 71.01% | 62.77% | 81.72% | 71.62% |
| **ALL** | 54.24% | 75.80% | 64.12% | 57.50% | 77.37% | 66.70% | 52.75% | 81.92% | 65.74% |

1. **Intersection call set of panSV and manta on HG002 148 dataset**

|  | **126×2 Illumina** | | | **148×2 Illumina** | | | **250×2 Illumina** | | |
| --- | --- | --- | --- | --- | --- | --- | --- | --- | --- |
|  | **Sensitivity** | **Precision** | **F1 score** | **Sensitivity** | **Precision** | **F1 score** | **Sensitivity** | **Precision** | **F1 score** |
| **INS** | 11.73% | 96.36% | 33.62% | 14.91% | 97.38% | 38.11% | 9.10% | 96.59% | 29.64% |
| **DEL** | 38.40% | 95.84% | 60.66% | 42.43% | 95.93% | 63.80% | 40.31% | 96.71% | 62.44% |
| **ALL** | 22.74% | 96.00% | 46.73% | 26.32% | 96.41% | 50.37% | 22.03% | 96.68% | 46.15% |


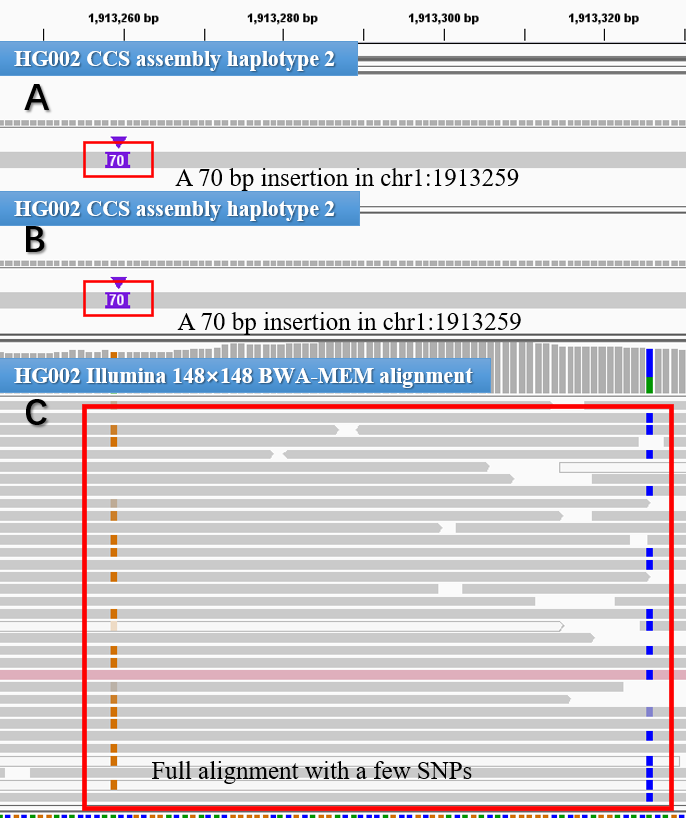


# Supplementary figure 1. The IGV result of a 70 bp insertion around chr1:1913259

Around chr1:1913259, BWA-MEM fully mapped nearly all short reads to reference with a few mismatches and did not generate any split read or discordant read pair signals. While panSVR can map those reads to pangenomes with higher alignment scores and detect SV. (A-B) Minimap2 aligning results of HG002 CCS assemblies on hs37d5(haplotype 1 and 2, respectively). (C) BWA-MEM aligning result of HG002 illumina 148×148 short reads on hs37d5.


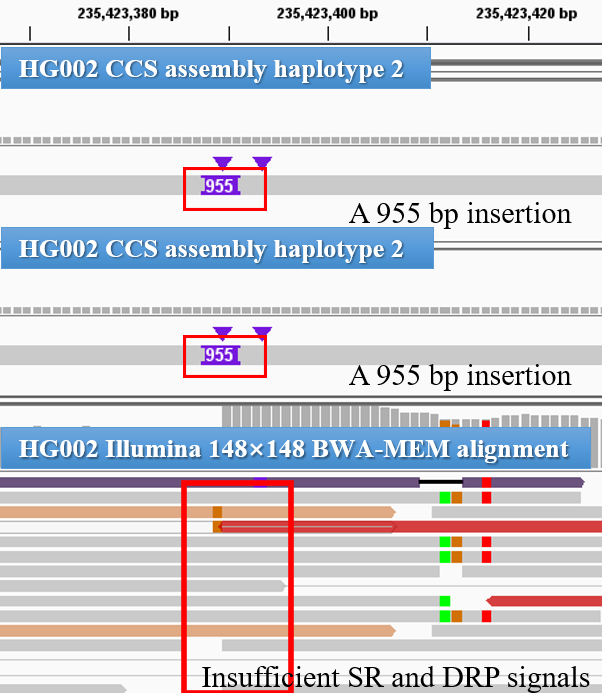


# Supplementary figure 2. The IGV result of a 955 bp insertion around chr2:235423389

Around chr2:235423389, panSV found a 955 bp long insertion, which was consistence with the SVIM-asm SV calling results on PacBio CCS assembly data. There is insufficient split read(SR) and discordant read pair(DRP) signals for downstream callers to call this insertion. (A-B) Minimap2 aligning results of HG002 CCS assemblies on hs37d5(haplotype 1 and 2, respectively). (C) BWA-MEM aligning result of HG002 illumina 148×148 short reads on hs37d5.


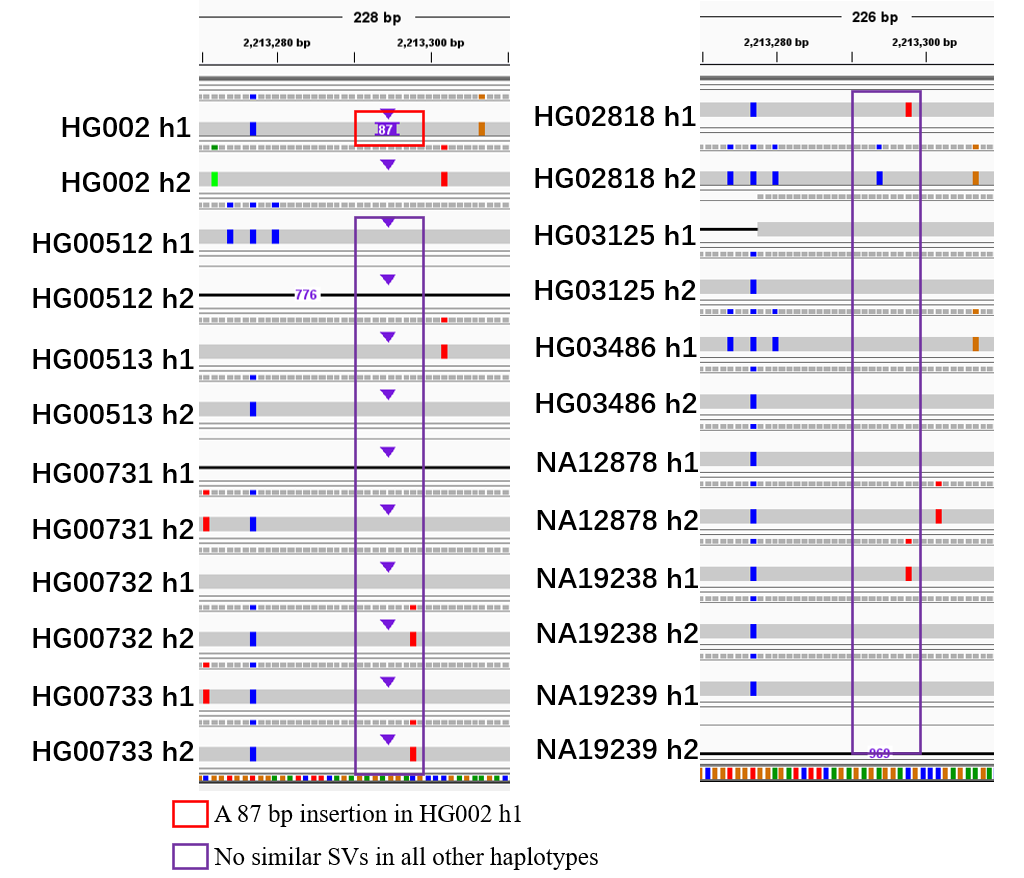


# Supplementary figure 3. The IGV result of an 87 bp SV around chr1: 2213294

There is an 87 bp SV in the first haplotype of HG0002 around chr1: 2213294. It is a singleton SV which does not appear in other samples. PanSVR failed to recall it.


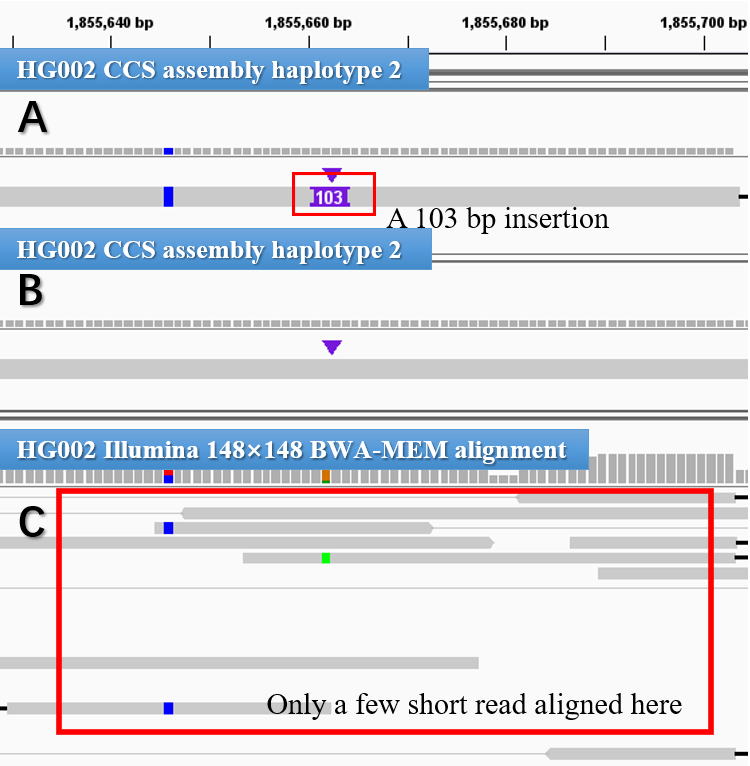


# Supplementary figure 4. The IGV result of a 103 bp SV around chr1: 1855662

The NGS reads of HG002 148×148 dataset have poor coverage around position chr1: 1855662. The inserted sequence is highly repetitive there, and short reads are usually mapped to other copies of the sequences with nearly perfect alignments. Therefore, PanSVR failed to recall that SV. (A-B) Minimap2 aligning results of HG002 CCS assemblies on hs37d5(haplotype 1 and 2, respectively). (C) BWA-MEM aligning result of HG002 illumina 148×148 short reads on hs37d5.

Command lines used in benchmark implementation.

(a) Building SV reference database.

First, the minimap2 (version 2.17-r974-dirty) hs37d5 index is building using this command:

minimap2 -x asm20 -d hs37d5.fa.mmi hs37d5.fa

Then, we align CCS assemblies on hs37d5 by minimap2 using following command, file name “sampleN_hx.fasta” is refer to the CCS assemble FASTA files for sample N and haplotype x.

minimap2 -a hs37d5.fa.mmi sampleN_hx.fasta | samtools view -b -o sampleN_hx.bam -

Next, all alignment results is sorted using samtools:

samtools sort --output-fmt=BAM -o sampleN_hx.sort.bam sampleN_hx.bam

Then, SVIM-ASM(ver 1.0.2) were used to call SVs for each sample:

svim-asm diploid sampleN_h1.sort.bam sampleN_h2.sort.bam hs37d5.fa

Then, all sample VCFs (except the one used for leaving-one-out) will be merged using SURVIVOR (ver 1.0.7):

ls *.vcf > merge_fn_list.txt

SURVIVOR merge merge_fn_list.txt 50 1 1 1 0 50 merge.vcf

Finally, SV reference will be generate using PanSV:

panSV sv_ref -N -b -e 200 hs37d5.fa merge.vcf > sv_ref.fa 2> sv_ref.log

(b) Benchmark dataset and ground truth

First, the original BAM/CRAM files will be sorted by read name using samtools.

samtools sort --output-fmt=BAM -n --reference hs37d5.fa(or GRCH38) -o sort_name.bam original.cram

Then, original FASTQ will be retrieved from read name sorted BAM files using samtools fastq module):

samtools fastq -c 4 -1 1.fastq.gz -2 2.fastq.gz sort_name.bam

Next, all FASTQ files will be alignment using BWA-MEM.

bwa mem -t 16 hs37d5.fa 1.fastq.gz 2.fastq.gz | samtools view -o BWA.hg37d5.sort_name.bam -

Finally, BAM files will be sort and index using samtools.

samtools sort --output-fmt=CRAM --reference hs37d5.fa -o BWA.hg37d5.sort_pos.bam BWA.hg37d5.sort_name.bam

samtools index BWA.hg37d5.sort_pos.bam

(c) Command lines used for SV callings.

1) Benchmark for PanSV.

Generate SV reference:

panSV sv_ref -N -b -e 200 hs37d5.fa merge.vcf > sv_ref.fa 2> sv_ref.log

Building deBGA index(“./deBGA_index” is a directory to store index):

deBGA index -k 22 sv_ref.fa ./deBGA_index

Read signal extraction (“hs37d5.header” is a tmp file generated here and used at following steps):

panSV signal -D -U BWA.hg37d5.sort_pos.bam -H hs37d5.header | pigz -p 8 -- > signal.fastq.gz

Alignment of read signals and sort by pos:

panSV aln -o panSV.signal.bam /deBGA_index signal.fastq.gz hs37d5.header

samtools sort -b -o panSV.sort.bam panSV.signal.bam

Generate SVs, the final result stored in panSV.vcf:

panSV assembly -D -S 0 -s 0 -E 24 -F 300000000 -e 200 -M 80 ./deBGA_index panSV.sort.bam hs37d5.header. hs37d5.fa > panSV.SV.log 2> panSV.vcf

2) Benchmark for Manta (ver 1.6.0).

Config for manta:

./configManta.py --referenceFasta=hs37d5.fa --bam BWA.hg37d5.sort_pos.bam

Running manta SV caller:

./runWorkflow.py -j 1

3) Benchmark for delly (ver 0.8.6).

./delly call -g hs37d5.fa -o delly.bcf BWA.hg37d5.sort_pos.bam

(d) Command lines used for comparing with the ground truth.

Separate SVs by type and discard SVs in decoy sequences:

panSV analysis vcf_dump ALL INS ALL caller.vcf > caller.type.vcf

panSV analysis vcf_dump ALL DEL ALL caller.vcf > caller.type.vcf

Compare call results using SURVIVOR:

mkdir ./caller_benchmark_folder & cd ./caller_benchmark_folder

mv ground_truth.type.vcf ./

mv caller.type.vcf ./

ls *.vcf > fn.txt

SURVIVOR merge ./fn.txt 1000 1 1 1 0 50 ./merge.vcf

SURVIVOR genComp 1 ./merge.vcf
